# Report of a Case of Shoulder Presentation

**Published:** 1856-08

**Authors:** R. T. Foote

**Affiliations:** Society Hill, Alabama


### ARTICLE III.

*Report of a Case of Shoulder Presentation.* By R. T. Foote, M. D., of Society Hill, Alabama.

At 2 o'clock, A. M., on January 13th last, I was called to see a negro woman, belonging to Mr. H. G——, distant four miles. On arriving, I was informed by her mistress, that she was between 8 and 9 months gone with child, and was attacked about midnight with labor pains. Upon examination, could not make out the presentation—cervix high in the pelvis, and dilated to the size of a quarter of a dollar—pains seldom, with little force. Ordered a purgative dose of ol. ricini, and retired to rest. 8 o'clock, A. M., medicine operated, pains almost ceased; gave her 20 drops tinct. opii. and left, with directions to send for me when her pains became strong.

January 22d, 3 o'clock, P. M.—Sent for in haste to the same woman; on reaching her bed-side, I plainly saw labor had set in. Her owner informed me that she had been up and doing tolerable well from the time I left her before, until a few hours previous to my being sent for.

Upon examination, I found the os dilated very extensively, through which was protruded the membranes, containing an unusual quantity of liquor amnii. Still, could not determine the presentation; though, satisfied it was an abnormal one, or in the common phrase of the lying-in chamber, "all was not right." This gave me some uneasiness, and as the pains were active, I very soon made another examination. As I introduced my fore finger of the right hand within the vagina, the membranes ruptured, and most of the liquor amnii escaped; and much to my regret, found the right hand and arm in the vagina. I at once recognized a Shoulder Presentation, returned the hand and arm as far as possible, within the uterus, and kept it there, hoping, when uterine contraction again came on, that the head might descend. Notwithstanding my best efforts to prevent it, at every pain, the hand and arm would partly descend and head remain stationary.

I then determined on version. After the necessary preparations, I introduced my hand during the interval of pains, and succeeded, without any difficulty, in finding and bringing down one foot, (which I took care to secure,) the pains coming on at the same instant, the uterus contracted with so much violence, I was unable to turn the child. I then informed the owner of the woman her situation, as well as I could, and requested a consultation. A messenger was dispatched in haste for a neighboring practitioner, who came as soon as the distance would allow.

I returned to her bed-side, found her calm and quiet. Upon examination I found the foot and hand still occupying the upper portion of the vagina. I then cautiously returned the hand within the uterus, and gave the presenting foot a quick pull, at the same moment the uterus began to act, and to my great satisfaction, found the foot descending considerably, and the hand entirely out of reach. After another pain or two, by continuing these manipulations, I had the satisfaction of knowing, that the version was completed, and as I then had a foot presentation, the completion of labor was left to Nature. The pains continued good, and in 20 minutes the woman was delivered of a still-born child, weighing about nine pounds. By persevering in the usual remedies in such cases, it was restored to animation, and is now, 9th of April, a fine, healthy child. The woman recovered without any untoward symptoms. She is the rise of forty years old, and the mother of 13 children.

So, when brother Chip arrived, we had nothing to do but turn our faces homeward, and move off at the rate of eight miles an hour to bring us to our firesides in half the time, and for my part, I felt, after a thorough warming, I was willing to retire to rest for the remainder of the night; not wishing to be aroused from my slumber by the familiar sound, "Halloo, halloo, is the Doctor at home?" which so often salutes the ear of the faithful, and often wearied physician, during his few snatched moments of rest, to be hurried to the bed-side of some poor woman, suffering the pains of child-bearing, and again run the risk of encountering this, or some other of the family of abnormal presentations, from which I might not escape so well.

